# An integrated analysis of SLC7A11 as a pan-cancer immunotherapeutic biomarker with experimental validation of its regulation by miR-148b-3p in breast cancer

**DOI:** 10.3389/fimmu.2026.1752767

**Published:** 2026-05-19

**Authors:** Songchen Zhao, Miao Zheng, Guannan Ma, Kaifeng Niu, Lei Zhang

**Affiliations:** 1 Department of Medical Oncology, Shanghai East Hospital, School of Medicine, Tongji University, Shanghai, China; 2 The First Affiliated Hospital of Jinzhou Medical University, Jinzhou, China; 3 Zhejiang Key Laboratory of Digital Technology in Medical Diagnostics, Hangzhou, China; 4 China National Center for Bioinformation, Beijing, China; 5 Beijing Institute of Genomics, Chinese Academy of Sciences, Beijing, China

**Keywords:** SLC7A11, miR-148b-3p, ferroptosis, immune infiltration, immunotherapy, pan-cancer, breast cancer

## Abstract

**Background:**

Identifying reliable biomarkers for immunotherapy response remains challenging. Solute carrier family 7 member 11 (SLC7A11), a key regulator of redox homeostasis and ferroptosis, demonstrates significant tumor-promoting potential across cancers, yet its pan-cancer associations with immunotherapy-related features and interaction with microRNAs in triple-negative breast cancer pathogenesis remain elusive.

**Methods:**

We systematically analyzed SLC7A11 expression across cancer types using TCGA and GTEx data, and evaluated its associations with immunotherapy-related features. Survival analyses were assessed in general and immunotherapy-treated cohorts. A miRNA regulatory network targeting SLC7A11 was constructed in breast cancer and the SLC7A11-miR-148b-3p axis was tested via *in vitro* experiments in triple-negative breast cancer (TNBC) cell models (MDA-MB-231 and MDA-MB-468).

**Results:**

Pan-cancer analysis revealed upregulation of SLC7A11 transcriptional expression in over 88% tumor types, and associations with established immunotherapy-related features, including tumor mutational burden, microsatellite instability, PD-L1 expression, and immune infiltration. Higher SLC7A11 expression was associated with improved response to immunotherapy, but inferior prognosis in most tumors, including breast cancer. In TNBC cell models, *in vitro* experiments supported that miR-148b-3p negatively regulated SLC7A11, and higher SLC7A11 expression was associated with increased glutathione levels, reduced lipid peroxidation, and enhanced cell survival under ferroptosis-inducing conditions.

**Conclusions:**

Our pan-cancer analyses reveal a potential biological link between SLC7A11, ferroptosis, and tumor immunity, generating the hypothesis that SLC7A11 may serve as a biomarker for immune checkpoint inhibitor response. In TNBC models, we identify the miR-148b-3p/SLC7A11 axis as a potential regulator of ferroptosis resistance based on *in vitro* findings, warranting further *in vivo* validation.

## Introduction

Moderate efficacy of immune checkpoint inhibitors has been highlighted in a pan-cancer study of response across 27 cancer types (1). Predictive markers for response to immune checkpoint inhibitors includes tumor mutational burden (TMB), programmed cell death ligand 1 (PD-L1) expression, microsatellite instability (MSI), transcriptional signatures, tumor-infiltrating immune cells, homologous recombination deficiency (HRD), etc (2, 3). However, nearly 37% of cancer types demonstrated an overall response rate  ≤ 10% (1). Therefore, identification of biomarkers predictive of the response is still a major challenge of effective implementation.

Solute carrier family 7 member 11 (SLC7A11) is a multi-pathway transmembrane protein that mediates the uptake of extracellular cystine in exchange for glutamate (4). Cystine is then reduced to cysteine, a rate-limiting precursor for glutathione synthesis, protecting cells from oxidative stress. This process plays a crucial role in cell growth, proliferation, and metabolism (5). As an amino acid transporter, the SLC7A11-glutathione (GSH) system is a key cellular mechanism for defending against ferroptosis (6). Inhibiting SLC7A11 expression disrupts the cystine metabolism pathway, leading to reduced intracellular cystine levels and depletion of GSH biosynthesis. This indirectly inhibits GSH peroxidase 4 (GPX4) activity, resulting in lipid peroxide accumulation and ultimately inducing ferroptosis (7). SLC7A11 is not only a potent target of ferroptosis but also plays an important role in a newly discovered form of cell death called disulfidptosis (8). Recent studies have shown that in tumors with high SLC7A11 expression, glucose deprivation leads to insufficient NADPH supply from the pentose phosphate pathway, causing a buildup of cystine that cannot be reduced to GSH. This accumulation of disulfides leads to cytoskeleton collapse, a characteristic of disulfidptosis, which can be induced by glucose transporter inhibitors and thiol-oxidizing agents like diamide (9).

Previous studies employed diverse regulators of SLC7A11 to modulate its expression or activity to influence ferroptosis (10–12). MicroRNAs micro-373 and -372 have been demonstrated to upregulate SLC7A11 expression through competitive binding, thereby regulating immune infiltration in lung adenocarcinoma. Inhibiting SLC7A11 selectively kills KRAS-mutant lung adenocarcinoma cells and inhibits tumor growth *in vivo* (13). In melanoma, SLC7A11 increases intracellular glutathione levels, conferring resistance to BRAF inhibitors (14). In bladder cancer, inhibiting SLC7A11 expression reverses cisplatin resistance in resistant cells (15). However, studies on whether and how SLC7A11 works with microRNAs to mediate ferroptosis in breast cancer are lacking. The association of SLC7A11 expression with immunological features and response to immune checkpoint inhibitors therapy across various tumor types remains elusive. While previous pan-cancer studies have primarily focused on SLC7A11’s prognostic value, a comprehensive integration of immune and ferroptosis analyses with mechanistic validation in specific cancer contexts remains lacking.

Here, a systematic and in-depth analysis of SLC7A11 expression was performed to examine association between SLC7A11 expression and various immunological features related to immunotherapy across tumor types, especially breast cancer. We further used *in vitro* experiments to elucidate how SLC7A11 and miR-148b-3p interacted to governs ferroptosis resistance in triple-negative breast cancer (TNBC) cell models.

## Materials and methods

This study was exempted from approval by an institutional review board and from the need for informed consent because its data were gathered from publicly available data sets that have received approval.

### Acquisition of SLC7A11 mRNA expression data and differential analysis across various tumor types

Based on RNA-seq expression data across 30 tumor types from The Cancer Genome Atlas (TCGA), we first utilized the TIMER2.0 (https://compbio.cn/timer2/) to perform differential test of SLC7A11 mRNA expresion between tumor and normal tissues. Normal samples from the TCGA are adjacent to the tumor and typically limited in number. Their proximity to tumor may introduce signals of tumor microenvironment in their SLC7A11 expression profile. Thus, we also retrieved SLC7A11 mRNA expression data of non-diseased tissues in Genotype-Tissue Expression (GTEx) from UCSC Xena (https://xena.ucsc.edu), where TCGA and GTEx data were co-analyzed by the same Toil RNA-seq pipeline to eliminate computational batch effects. The expression values of SLC7A11 were quantified by RNASeq by Expectation-Maximization algorithm and then normalized using the upper quartile method. The normalized values were log2-transformed after adding an offset of 0.001 to avoid taking log of zero before analysis. Differential expression analysis of SLC7A11 based on combined TCGA and GTEx data was performed using the R package limma.

### Acquisition of SLC7A11 protein expression data across various tumor types

Pan-cancer protein expression dataset was retrieved from the Clinical Proteomic Tumor Analysis Consortium (CPTAC). Upon inspecting this dataset, SLC7A11 protein expression data was available for three tumor types, including lung squamous cell carcinoma (LUSC), head and neck squamous cell carcinoma (HNSC), and glioblastoma (GBM). The log2 spectral count ratio values from the datasest were first normalized within each sample profile, then normalized across samples. Z-values of SLC7A11 protein expression represent standard deviations from the median across samples for the given cancer type. Lastly, the UALCAN (http://ualcan.path.uab.edu) was utilized to examine differential expressions of SLC7A11 protein between tumor and normal tissues.

### Immunotherapy-related genomic markers and methylation-related mRNAs

To investigate the association of SLC7A11 expression with response to immune checkpoint inhibitors therapy, we collected well-established genomic features related to the response for the TCGA samples. Specifically, based on the TCGA somatic mutation data, TMB was calculated as the total count of nonsynonymous mutations in coding sequence using the R package maftools. MSI and HRD data were downloaded from previous reports (16, 17). Given the clinical relevance of mismatch repair deficiency, we focused on Lynch syndrome-associated mismatch repair genes (*MLH1*, *MSH2*, *MSH6*, *PMS2*, and *EPCAM*) as commonly used markers of mismatch repair deficiency in pan-cancer studies (18).

Epigenetic modifications play crucial roles in cancer initiation and progression, emerging as important biomarkers to identify responders to immunotherapy. Notably, methylation plays a critical role in the ferroptosis process mediated by SLC7A11. Classical forms of such modifications regarding RNA include N6-methyladenine (m6A), 5-methylcytosine (m5C), and N1-methyladenine (m1A). Thus we retrieved 44 m1A/m5C/m6A-related genes (10, 13, and 21 genes, respectively) to investigate the relationship between RNA methylation and SLC7A11 expression (19).

### Immune-related genes expression profile and tumor immune microenvironment

We first collected 60 immune-related genes, which were classified into two categories: inhibitory and stimulatory. Their normalized expression levels were extracted for the TCGA tumor samples with both matched SLC7A11 expression data available, and then log2-transformed after adding an offset of 0.001 for subsequent correlation analysis. Next, we gathered six immune infiltrates (B cells, CD4^+^ T cells, CD8^+^ T cells, neutrophils, macrophages, and dendritic cells) of the TCGA tumor samples with their abundances estimated by TIMER algorithm (https://cistrome.shinyapps.io/timer/). Lastly, we evaluated the extent of immune cells infiltration into TIME by generating a composite immune score that represents the infiltration of immune cells in tumor tissue using the R package ESTIMATE. In addition, Previous study reported the correlation of cancer stemness with immune checkpoint expression and infiltrating immune cells (20), thus we downloaded RNA stemness score to examine whether SLC7A11 expression correlated with cancer stemness. RNA stemness score ranges from 0 to 1. A score close to 1 indicates the lower cell differentiation extent and stronger stem cell characteristics.

### Survival analysis and evaluation of diagnostic value

We first collected integrated curated survival data across pan-cancer from TCGA. Measurements of survival outcomes comprised overall survival (OS), disease-specific survival (DSS), disease-free interval (DFI), and progression-free interval (PFI), aiming to comprehensively explore prognostic value of SLC7A11 expression in each tumor type. Kaplan-Meier curves were generated, and differences between groups were evaluated using the log-rank test. Hazard ratios (HRs) with 95% confidence intervals (CIs) were estimated using Cox proportional hazards models.

To further examine the prognostic value of SLC7A11 in BRCA, we analyzed two BRCA cohorts with OS and SLC7A11 expression data available which were derived from manually curated KM-plotter database and GSE3494 dataset.

To investigate the association of SLC7A11 expression with response to immune checkpoint inhibitors, we compared the SLC7A11 expression in responders versus non-responders from previous studies receiving immune checkpoint inhibitors treatment (21). Response was based on Response Evaluation Criteria in Solid Tumors (RECIST) v1.1. Patients who experienced complete response or partial response were classified as responders; patients who experienced stable disease or progressive disease were classified as non-responders. Furthermore, potential value of SLC7A11 expression in tumor diagnosis was evaluated via receiver operating characteristic curve (ROC), with an area under the curve (AUC) > 0.8 considered to indicate high diagnostic value.

### Construction of SLC7A11-related MiRNA regulatory network

We investigated the miRNA regulatory network associated with SLC7A11 in BRCA. Upstream miRNAs potentially binding to SLC7A11 were predicted using multiple target gene prediction tools, including PITA, RNA22, miRmap, microT, miRanda, PicTar, and TargetScan. To ensure reliability, only miRNAs predicted by at least three tools were selected. The miRTarBase (https://mirtarbase.cuhk.edu.cn) collected experimentally validated microRNA-target interactions. Thus, miRNA predictions targeting the SLC7A11 gene were cross-referenced using the ENCORI (https://rna.sysu.edu.cn/encori) and miRTarBase platforms. The overlap was designated as candidate miRNAs for SLC7A11. Given that mRNA levels are expected to be inversely correlated with miRNA levels, correlations between candidates and SLC7A11 expression were assessed using the ENCORI to construct the miRNA regulatory network in the BRCA cohort. The ENCORI utilizes expression data derived from the TCGA project.

### Acquisition of ferroptosis-related genes data

STAR-counts data and corresponding clinical information for BRCA were downloaded from the TCGA database. Then we extracted expression data normalized in transcripts per million (TPM) format, following the log2(TPM + 1) transformation. The ferroptosis-related genes were derived from a systematic analysis of the abnormalities and functions of ferroptosis in cancer by previous work (22).

### Cell culture and antibodies

TNBC cell models MDA-MB-231 and MDA-MB-468 were obtained from the American Type Culture Collection (ATCC). MDA-MB-231 cells were cultured in DMEM, while MDA-MB-468 cells were maintained in RPMI 1640 medium (Gibco, Thermo Fisher Scientific). Both media were supplemented with 10% fetal bovine serum (Gibco, Thermo Fisher Scientific). Cells were incubated at 37 °C in a humidified atmosphere containing 5% CO_2_. Mycoplasma was tested as negative using Mycoplasma Detection Kit (Biotool, USA). Following antibodies were used: SLC7A11 (A2413, Abclonal) and GAPDH (MB374, Millipore).

### Glutathione assay

The GSH assay was conducted using Total Glutathione Assay Kit (S0052, Beyotime) following the manufacturer’s instructions. Briefly, MDA-MB-231 or MDA-MB-468 cells were transfected with control or miR-148b-3p inhibitors for 24 hours, followed by SLC7A11 overexpression, and then treated with 10 μM erastin (329600, Sigma-Aldrich) for 5 hours, and the intracellular GSH level were measured according to the manufacturer’s instructions. The total GSH content can be calculated by measuring 412 nm (A412).

### Assessment of lipid peroxidation by measuring malondialdehyde

MDA-MB-231 or MDA-MB-468 cells were transfected with control or miR-148b-3p inhibitors for 24 hours, followed by SLC7A11 overexpression, and then treated with 10 μM erastin (329600, Sigma-Aldrich) for 5 hours, and the intracellular malondialdehyde (MDA) level were measured using Lipid Peroxidation MDA Assay Kit (S0131M, Beyotime) following the manufacturer’s instructions.

### Proliferation measurement of MDA-MB-231 cells

Cell Counting Kit-8 (CCK‐8) kit (C0042, Beyotime) was used to measure proliferation of MDA-MB-231 cells according to the manufacturer’s protocols. 2000 cells with or without miR-148b-3p inhibitors were seeded into each well of 96-well plates and incubated for 1, 2, 3, and 4 days. 10 μL of CCK‐8 reagent was added to each well at indicated time points and then cultured for 1 hour in a CO_2_ incubator (5% CO_2_) at 37 °C. The absorbance was analyzed at 450 nm using a microplate reader (Bio‐Rad, USA) using wells without cells as blanks. The proliferation of cells was expressed by the absorbance. In total ≥ six control wells were included for each time point in all experiments. All experiments were performed in triplicate.

### Migration by MDA-MB-231 cells

Migration by MDA-MB-231 cells with or without miR-148b-3p inhibitors was tested using transwell assays. 5000 cells per well were seeded into each well of 24-well transwell chambers (ECM508, MilliporeSigma) and incubated at 37 °C in 5% CO_2_ for 48 hours. The migrated cells were stained with cell stain reagent for 20 minutes and extracted using extraction buffer to measure the level of optical density at 560 nm. Images were captured using a Leica DM5000 microscope.

### Statistical analyses

Statistical significance of differential expression between two groups was evaluated using Wilcoxon rank-sum test unless otherwise specified. Correlation between expression of genes was estimated using Pearson correlation analysis. All experimental data based on cells were presented as the mean ± standard deviation (SD) from three independent biological experiments, and their differences between groups were compared using a Student’s t-test. Adjusted *P* values were calculated using the false discovery rate method (FDR) and provided in supplementary tables. All statistical analyses above were done using GraphPad Prism 8.0 software. Statistical significance was set at two-tailed FDR-adjusted *P* < 0.05 unless otherwise specified.

## Results

### SLC7A11 transcriptional expression elevated in majority of tumors

**Figure 1:**
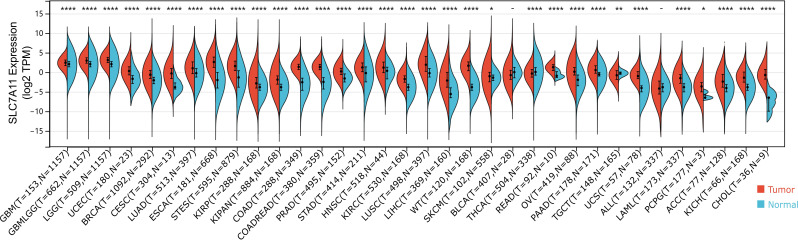
Comparison of mRNA expression pattern of SLC7A11 in normal versus tumor tissues. The violin plot illustrates the mRNA expression levels of SLC7A11 (log2 Transcripts Per Million, TPM) in various tumor tissues (red) compared to their corresponding normal tissues (blue) using data from TCGA and GTEx databases. Each violin represents the density distribution of expression, with the center black dot indicating the median and whiskers representing the confidence interval. The sample sizes for tumor (T) and normal (N) cohorts are indicated below each cancer abbreviation. Statistical significance was determined using Wilcoxon rank-sum test, with adjusted p-values indicated as follows: *P < 0.05, **P < 0.01, and ****P < 0.0001; -, not significant

We investigated SLC7A11 mRNA expression differences in normal versus tumor tissues based on combined TCGA and GTEx data. The results revealed significant differences in the transcriptional level across pan-cancer. Specifically, we found significant upregulation in 88.24% (30/34) of tumors and downregulation in 5.88% (2/34) (FDR-adjusted *P* < 0.05), with BLCA and ALL being the exceptions (**Figure 1**). The similar trend was also observed in the TCGA data (**Supplementary Figure 1**). We further examined whether there were similar expression pattern in terms of protein abundance using the CPTAC proteomics data accessed via the UALCAN portal. Increased SLC7A11 protein levels were observed in HNSC and LUSC, whereas GBM showed decreased protein abundance (**Supplementary Figure 2**). Overall, the directionality of protein-level changes was not uniform across the available CPTAC tumor types. Such mRNA-protein discordance is not uncommon and may reflect tumor-type-specific post-transcriptional regulation, differences in protein turnover, tumor purity, and platform-related factors; therefore, the proteomic results are presented as supportive evidence and should not be overgeneralized across cancers.

### SLC7A11 expression associated with TMB and other established markers for immune checkpoint inhibitors therapy

To investigate association of SLC7A11 expression with well-established genomic factors for response to immune checkpoint inhibitors, we began with tumor mutational burden (TMB), because it has been the most widely reproduced immunotherapy biomarker. We found statistically significant positive association between SLC7A11 expression and TMB across multiple tumors, with Pearson’s coefficient (r) ranging from 0.08 to 0.31 (FDR-adjusted *P* < 0.05), including BRCA, THYM, ACC, COAD, COADREAD, UCEC, and STAD (**Supplementary Figure 3A**). The strongest association was observed in THYM (r = 0.31). Next, the association between SLC7A11 expression and microsatellite instability (MSI) status was evaluated, and positive correlations were observed across 7 tumor types with r ranging from 0.16 to 0.34 (FDR-adjusted *P* < 0.05) (**Supplementary Figure 3B**). Additionally, homologous recombination deficiency (HRD) is also a common driver of genomic instability and confers a therapeutic vulnerability in multiple cancers (23, 24). Our results showed that there were significant correlations between SLC7A11 expression and HRD across 14 tumor types, with r ranging from 0.11 to 0.37 (FDR-adjusted *P* < 0.05), including BRCA (r = 0.18) (**Supplementary Figure 3C**).

The MMR mechanism is intricate, designed to ensure genomic stability and integrity by detecting and correcting abnormal sequences and structures within chromosomes. Tumor cells often exploit the MMR pathway to evade treatment, gaining self-renewal capabilities similar to stem cells. Therefore, we explored the potential connections between SLC7A11 expression and MMR-related genes (*PMS2*, *MSH6*, *MSH2*, *MLH1*, and *EPCAM*), as well as tumor stemness. Notably, the expression of SLC7A11 significantly correlated with at least one MMR-related genes across various tumor types, with strongest correlations observed in BRCA between SLC7A11 and MSH6 (r = 0.38, FDR-adjusted *P* = 3.2×10^-36^) as well as MSH2 (r = 0.36, FDR-adjusted *P* = 1.1×10^-32^) (**Supplementary Figure 4A**).

Given reported correlation of cancer stemness with immune checkpoints expression and infiltrating immune cells, the correlation between the RNA stemness score and SLC7A11 expression was calculated. We observed significant positive correlations across 10 tumor types, with r ranging from 0.12 to 0.34 (FDR-adjusted *P* < 0.05), including BRCA, BLCA, ESCA, LAML, LUAD, PRAD, KIRP, STES, LUSC, and STAD (**Supplementary Figure 4B**).

### Association between SLC7A11 and genes involved in epigenetic modifications

We further analyzed the relationship between expression of SLC7A11 and 44 hallmark genes involved in three types of epigenetic modifications m1A, m5C, and m6A. As presented in **Supplementary Figure 4C**, SLC7A11 expression was observed to be positively correlated with epigenetic modification-related genes (FDR-adjusted *P* < 0.05) in most tumors, including BRCA with strongest correlation observed for the m5C writer TRDMT1 (r = 0.39). These results implied potential roles of SLC7A11 with regard to DNA methylation and mRNA modifications in the context of tumor pathogenesis.

### SLC7A11 expression associated with immune regulation genes and immune infiltration

Given the critical role of immune checkpoints in determining the efficacy of immunotherapy, we investigated potential correlations between SLC7A11 expression and 60 immune checkpoint pathway genes across various cancers. Interestingly, SLC7A11 exhibited widespread significant associations with immune inhibitory and stimulatory genes across various tumors, with positive correlations being predominant (**Supplementary Figure 5A**). In tumors like ESCA, significant associations were observed between the expression of SLC7A11 and almost all immune checkpoint pathway genes. Moreover, CD274 (PD-L1) significantly correlated with SLC7A11 expression in 30 tumor types, supporting the potential involvement of SLC7A11 in the anti-PD-L1 immunotherapy response. It is also noted that, well-established immune checkpoints showed strong positive associations with SLC7A11 expression in BRCA, such as PD-L1 (r = 0.23, FDR-adjusted *P* = 1.1×10^-12^). Taken together, these findings suggest that SLC7A11 expression is associated with multiple immunotherapy-related features, warranting further evaluation in well-annotated immune checkpoint inhibitors-treated cohorts.

**Figure 2:**
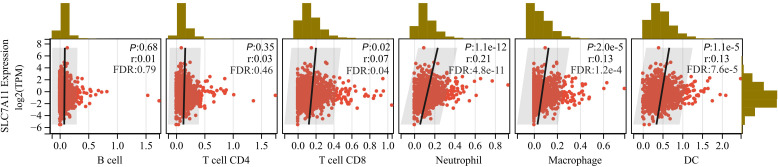
Correlation analysis between SLC7A11 expression and immune cell infiltration in breast cancer (BRCA). Scatter plots display the correlation between SLC7A11 mRNA expression levels and the infiltration levels of various immune cell types, including B cell, CD4^+^ T cell, CD8^+^ T cell, neutrophil, macrophage, and dendritic cell (DC), based on the TCGA-BRCA dataset. The vertical axis represents the log2-transformed expression levels of SLC7A11, quantified as TPM (Transcripts Per Million) values, and the horizontal axis indicates the immune infiltration score. Marginal histograms show the distribution of each variable. The correlation coefficient (r) and statistical significance (*P*) were determined using Pearson correlation analysis. The FDR represents an adjusted p*-*values.

Given the link between SLC7A11 and immune-releated genes, we further assessed the relationship between SLC7A11 expression and immune cells infiltration into tumor immune microenvironment using the TIMER2. Specifically, six tumor-infiltrating immune cells types, including B cell, CD4^+^ T cell, CD8^+^ T cell, neutrophil, macrophage, and dendritic cell, were examined. We found that higher SLC7A11 expression indicated greater extent of CD8^+^ T cells, neutrophils, macrophages, and dendritic cells infiltration into tumor immune microenvironment for the majoriy of tumors (**Supplementary Figure 5B**). Additionally, the most pronounced positive correlation between immune cell infiltration and SLC7A11 was found for CD8^+^ T cell in THCA (r = 0.51, FDR-adjusted *P* = 2.9×10^-32^), whereas the strongest negative correlation was observed for CD8^+^ T cell in HNSC (r = -0.35, FDR-adjusted *P* = 7.7×10^-14^). To confirm the strongest association in HNSC, we generated a composite immune score based on immune cell types in innate and adaptive immune responses, and observed a significantly decreased immune score with increasing SLC7A11 expression (**Supplementary Figure 6**). Also, we found that significantly positive associations between SLC7A11 expression and infiltration extent of CD8^+^ T cell, neutrophil, macrophage, and dendritic cell persisted in BRCA, with r ranging from 0.07 to 0.21 (FDR-adjusted *P* < 0.05) (**Figure 2**).

### SLC7A11 expression associated with clinical outcomes across multiple tumor types including BRCA

**Figure 3:**
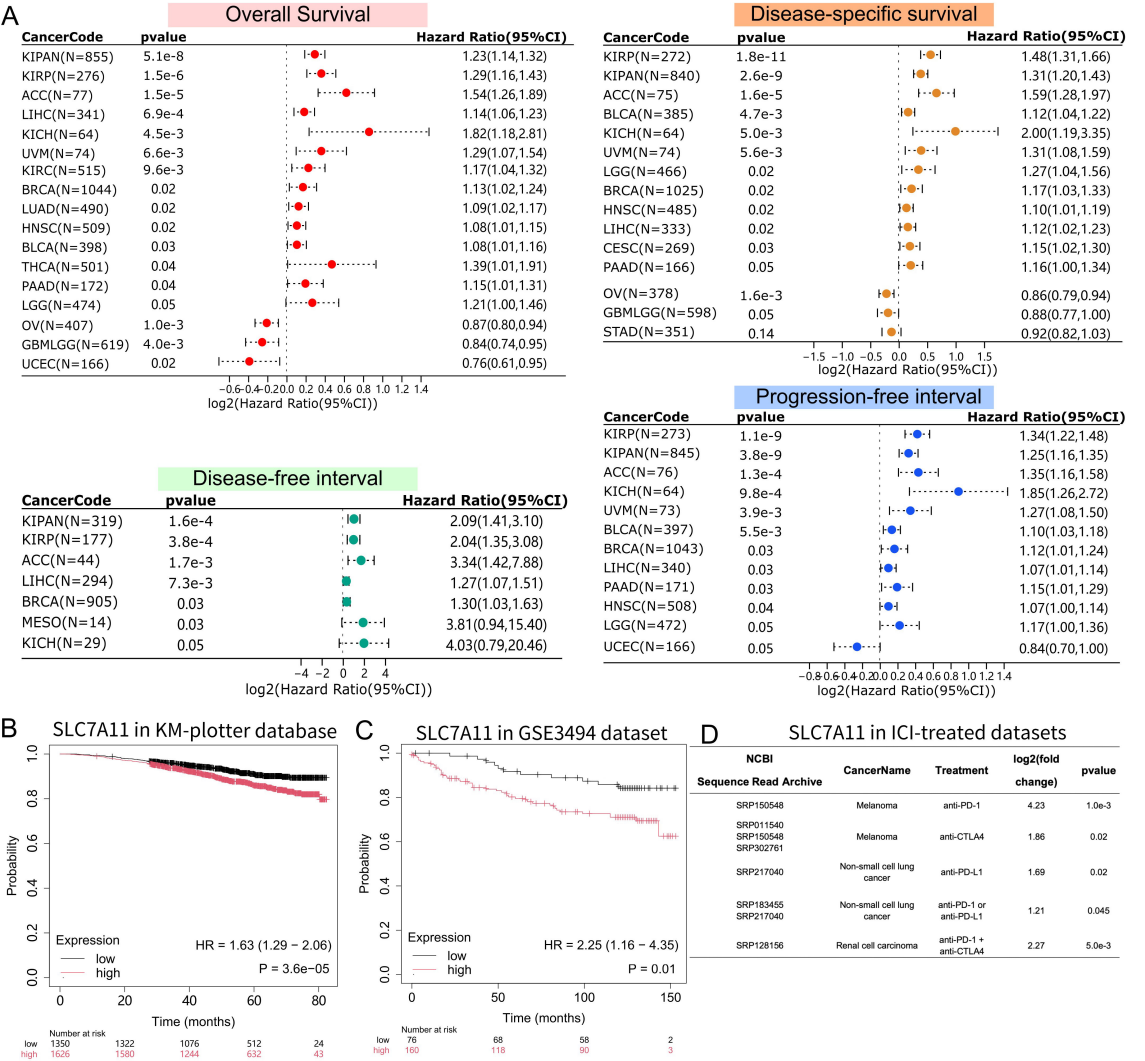
Association of SLC7A11 with outcomes across multiple datasets. **(A)** Forest plots summarizing the association between SLC7A11 expression and four clinical survival outcomes: overall survival, disease-specific survival, disease-free interval, and progression-free interval. Hazard ratios (HR), 95% confidence intervals (CI), and *P* values are shown. **(B, C)** Kaplan-Meier survival curves of SLC7A11. Survival analysis shows the association between SLC7A11 expression (high versus low) and patient probability of survival in the KM-plotter database **(B)** and the GSE3494 dataset **(C)**. The log-rank test was used to calculate statistical significance. **(D)** SLC7A11 expression in ICI-treated cohorts. The table summarizes the differential expression of SLC7A11 between responders and non-responders treated with various immune checkpoint inhibitors (ICI), including anti-PD-1, anti-PD-L1, anti-CTLA4, or combination therapy, across multiple NCBI Sequence Read Archive datasets. The fold change was calculated as the ratio of the mean expression level in the responders to that in the non-responders. Statistical significance was determined by t-test.

Based on the findings above, we further examined whether SLC7A11 expression was associated with patient prognosis. We collected clinical outcomes from TCGA and GTEx pan-cancer cohorts, including OS, DSS, PFI, and DFI. Survival analysis revealed that higher expression of SLC7A11 were associated with worse OS across 14 tumor types (**Figure 3A**). We further assessed the association of SLC7A11 expression with DSS, DFI, and PFI, and observed that higher SLC7A11 expression was linked to worse DSS in 14 tumor types, worse DFI in 7 types, and worse PFI in 11 types. Overall, elevated SLC7A11 expression was associated with poor outcome in 6 cancer types, including BRCA, KIPAN, KIRP, ACC, LIHC, and KICH (**Supplementary Figure 7A**). Intriguingly, ROC analyses demonstrated potential diagnostic value of SLC7A11 in certain cancers, such as KICH, KIRP, and ESCA (**Supplementary Figure 7B**).

Further validation analyses were performed to confirm the association of SLC7A11 expression with outcomes in other BRCA cohorts. In both cohorts, high expression of SLC7A11 was observed to be significantly associated with poor prognosis (cohort 1: HR = 1.63, *P* = 3.6×10^-5^; cohort 2: HR = 2.25, *P* = 0.01) (**Figures 3B, C**). Notably, we observed generally higher SLC7A11 expression in responders versus non-responders across several cancer types treated with immune checkpoint inhibitors, particularly in melanoma, as well as in renal cell carcinoma and non-small cell lung cancer (**Figure 3D**).

### MiR-148b-3p/SLC7A11 axis regulates ferroptosis resistance and malignant phenotypes in TNBC cells

**Figure 4:**
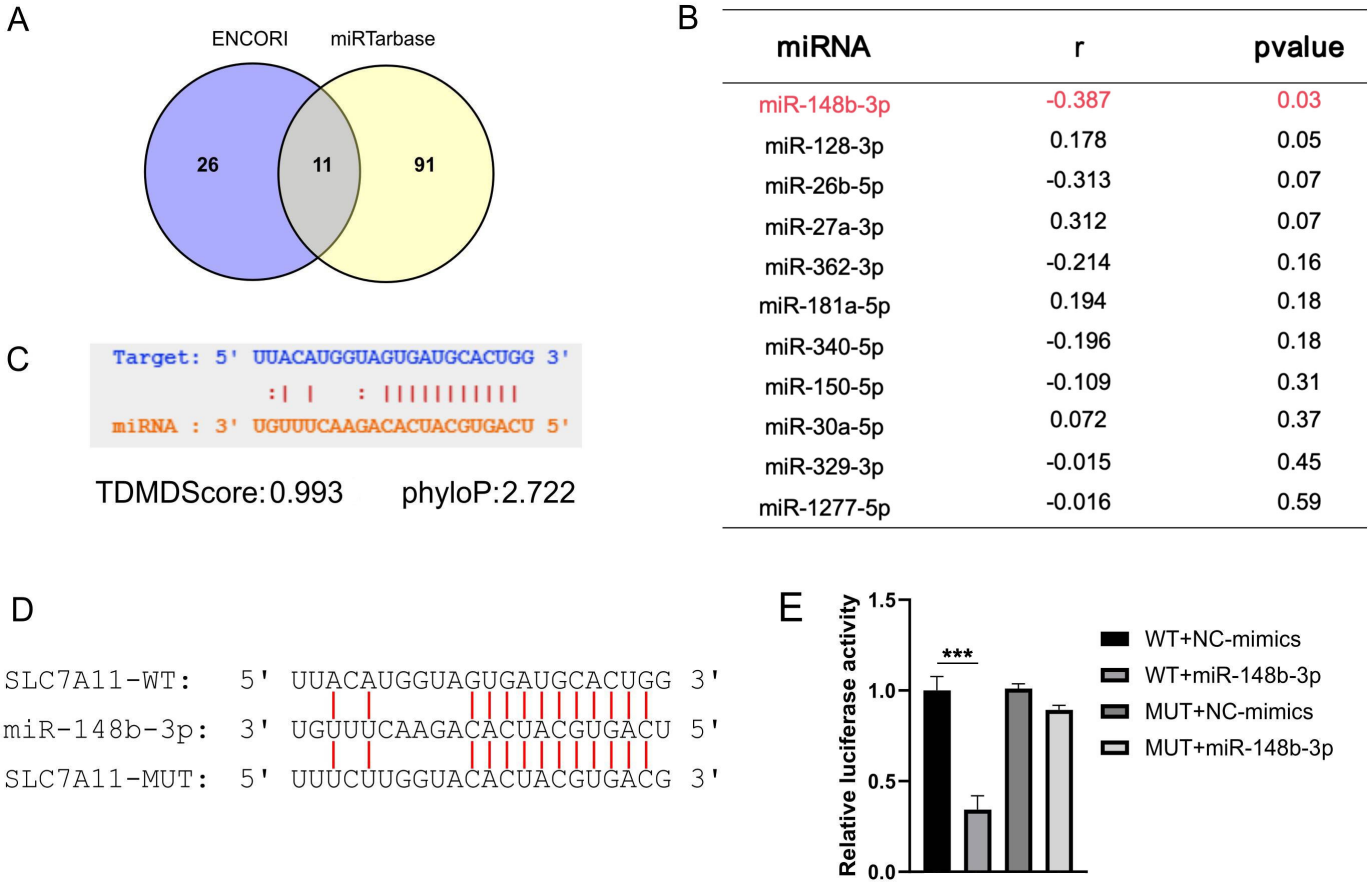
Identification and characterization of the miR-148b-3p/SLC7A11 regulatory axis. **(A)** Venn diagram illustrating the intersection of candidate miRNAs targeting SLC7A11 as predicted by ENCORI and miRTarBase databases. **(B)** Pearson correlation analysis between candidate miRNAs and SLC7A11 mRNA expression. MiR-148b-3p (highlighted in red) exhibited the most significant negative correlation (coefficient [r] = -0.387, *P* = 0.03). **(C)** Bioinformatic prediction via StarBase showing the putative binding site of miR-148b-3p within the SLC7A11 3’UTR. High evolutionary conservation (phyloP = 2.722) and strong potential for target-directed miRNA degradation (TDMDscore = 0.993) are indicated. **(D)** The sequences of wild-type (WT) and mutant (MUT) binding sites used for experimental validation. **(E)** The wild-type and mutated binding sites of SLC7A11 were cloned into the pGL3-control vector separately. HEK293 were transfected with the plasmids above and pRL-TK plasmids, followed by NC-mimics or miR-148b-3p mimics for 24 hours, and luciferase activities were measured. Data are presented as mean ± SD from three independent experiments. *** indicates *P* < 0.001.

Overexpression of SLC7A11 promotes cancer growth through suppressing ferroptosis in cancer cells (25). The involvement of competitive endogenous RNA (ceRNA) in tumorigenesis has been validated through various experiments. We focused on the upstream role of miR-148b-3p and showed that miR-148b-3p inhibition increased SLC7A11 expression, thereby affecting ferroptosis-related phenotypes. Firstly, we employed seven algorithms implemented in the ENCORI platform to identify potential miRNAs targeting SLC7A11, and then selected 37 miRNAs identified by at least three alorithms. Secondly, according to the curated miRTarbase database, there were 102 experimentally validated SLC7A11-targeting miRNAs, with 11 overlapping miRNAs observed (**Figure 4A**). Considering general negative correlation trend between miRNA and ceRNA (mRNA, lncRNA, etc.) expression, miR-148b-3p was selected (**Figure 4B**). MiRNA-target analysis confirmed a direct binding site for miR-148b-3p within the 3’-UTR of SLC7A11 (**Figure 4C**), and the luciferase reporter assay showed that miR-148b-3p mimics significantly decreased the luciferase activity of SLC7A11-WT, but not SLC7A11-mutant (MUT) (**Figures 4D, E**). These demonstrate that miR-148b-3p directly targets the 3’-UTR of SLC7A11.

**Figure 5:**
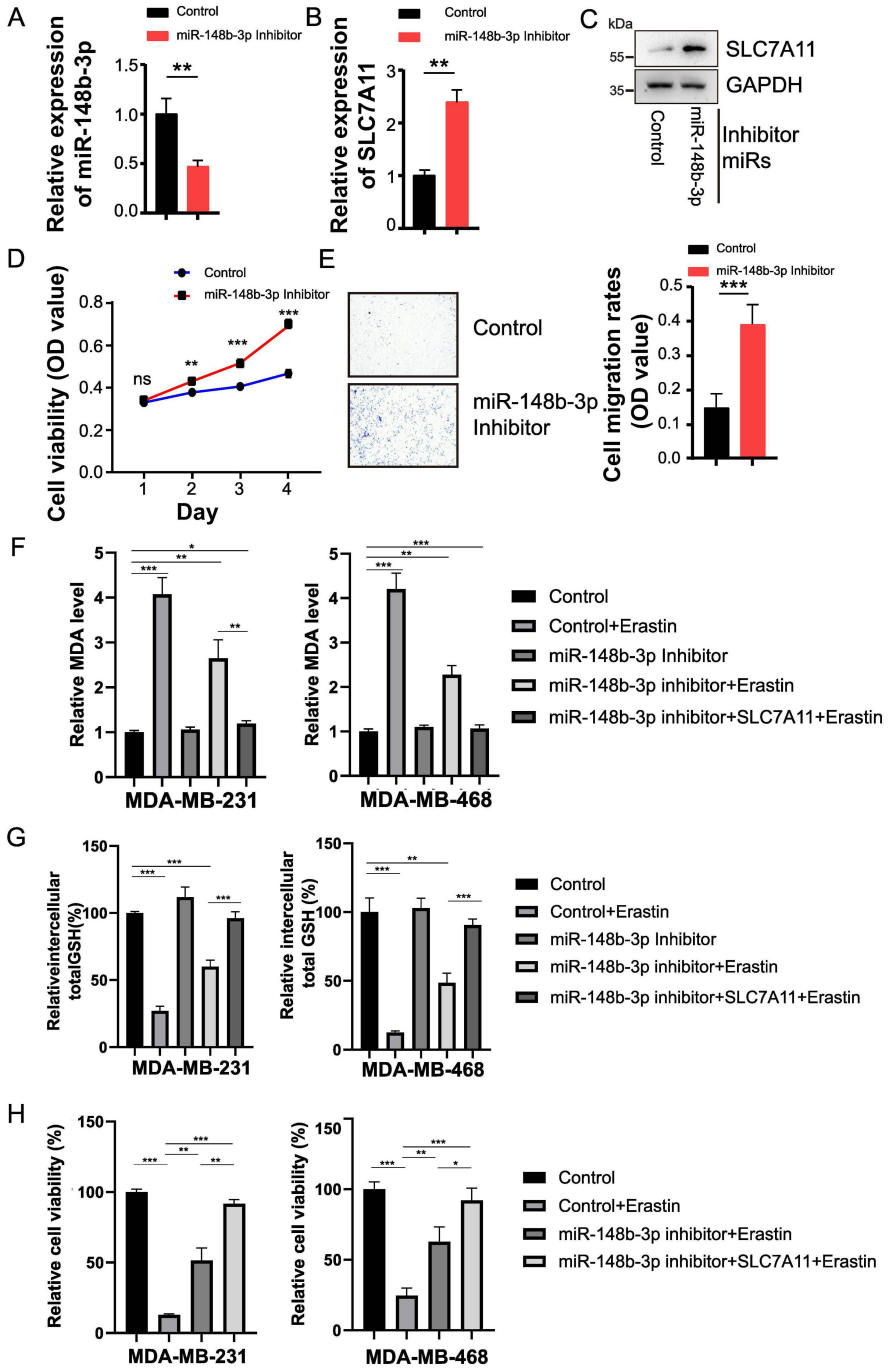
SLC7A11 promotes TNBC cell models progression and ferroptosis via regulation of miR-148b-3p. **(A)** Validation of miR-148b-3p knockdown efficiency by qRT-PCR, with U6 as an internal control. **(B, C)** Expression levels of SLC7A11 mRNA **(B)** and protein **(C)** were determined by qRT-PCR and western blotting, respectively, showing that miR-148b-3p inhibition significantly upregulated SLC7A11. GAPDH was used as the loading control. Uncropped western blot images are provided as **Supplementary Figure 8**. **(D)** MiR-148b-3p inhibitors or control were transfected into MDA-MB-231 cells. Cell proliferation was measured using CCK-8 assay. **(E)** Transwell assay was used for cell migration of MDA-MB-231 cells. **(F–H)** The miR-148b-3p/SLC7A11 axis modulated erastin-induced ferroptosis. MDA-MB-231 or MDA-MB-468 cells were transfected with control or miR-148b-3p inhibitors for 24 hours, followed by SLC7A11 overexpression, and then treated with 10 μM erastin. The MDA level **(F)**, GSH level **(G)**, and cell viability **(H)** were determined in indicated cells. Data are presented as mean ± SD from three biological replicates. * indicates *P* < 0.05, **P < 0.01, and ***P < 0.001; ns, not significant.

We anticipated that miR-148b-3p might play a crucial role in BRCA pathogenesis by targeting SLC7A11. To test this hypothesis, MDA-MB-231 cells transfected with miR-148b-3p inhibitors were collected 48 hours later. Real-time PCR results showed that miR-148b-3p inhibitors significantly reduced miR-148b-3p expression (**Figure 5A**). Silencing miR-148b-3p expression led to increased mRNA and protein levels of SLC7A11 (**Figures 5B, C**), supporting a regulatory relationship between miR-148b-3p and SLC7A11. A CCK-8 assay was used to determine cell viability at different proliferation stages. The results indicated that miR-148b-3p knockdown significantly enhanced cell viability compared to cells with negative control (NC) inhibitors (**Figure 5D**). Additionally, cell migration rate, as measured by the transwell assay, was significantly higher in cells transfected with miR-148b-3p inhibitors than that with NC inhibitors (**Figure 5E**). Overall, these findings suggest that miR-148b-3p inhibits proliferation and migration of TNBC cells by regulating SLC7A11.

To further elucidate the role of the miR-148b-3p/SLC7A11 axis in ferroptosis, TNBC cells were treated with erastin, a widely used ferroptosis inducer that functions by inhibiting the activity of system xc^-^. As shown in **Figures 5F–H**, erastin treatment significantly increased intracellular MDA levels (**Figure 5F**), decreased total intracellular GSH levels (**Figure 5G**), and reduced cell viability (**Figure 5H**) in MDA-MB-231 and MDA-MB-468 cells. Notably, inhibition of miR-148b-3p markedly reversed these effects, as evidenced by decreased MDA levels, increased GSH levels, and enhanced cell survival compared with the negative control group (**Figures 5F–H**). These findings suggest that miR-148b-3p regulates ferroptosis sensitivity through modulation of intracellular redox status. Functionally, re-expression of SLC7A11 further enhanced the observed phenotypic changes (**Figures 5F–H**), reinforcing its role in mediating ferroptosis resistance. Taken together, these findings indicate that inhibition of miR-148b-3p enhances resistance to erastin-induced ferroptosis through direct targeting of SLC7A11 in TNBC cells.

## Discussion

Our work advances beyond prior pan-cancer analyses of SLC7A11 by combining large-scale pan-cancer immune analyses in transcriptomic level with *in vitro* functional validation, focusing on the association of SLC7A11 with immunotherapy response and a regulator of ferroptosis via miR-148b-3p in TNBC cell models. Through pan-cancer analysis, we observed that SLC7A11 was frequently upregulated at the transcript level and was associated with poor prognosis in most tumors, consistent with its established role in promoting tumor growth and conferring resistance to various therapies (25–27). Relatedly, prior work has explored ferroptosis- and oxidative stress-based prognostic signatures in specific cancers (e.g., clear cell renal cell carcinoma), suggesting broader prognostic relevance of these pathways (28, 29). Intriguingly, we observed a paradoxical yet clinically significant pattern: higher SLC7A11 expression was associated with adverse prognosis in untreated patients, but with improved outcomes in immune checkpoint inhibitors-treated cohorts. Given the heterogeneity and limited clinical annotation of public immunotherapy datasets, this finding should be interpreted as association-based and hypothesis-generating observations.

In our pan-cancer analyses, higher SLC7A11 expression was associated with an inflamed tumor microenvironment in most tumors, characterized by elevated levels of immune checkpoint molecules and increased infiltration of cytotoxic CD8^+^ T cells, etc. This suggests that SLC7A11 may be associated with an immunogenic niche, potentially explaining the enhanced efficacy of immunotherapy in SLC7A11-high tumors. Additionally, this observation aligns with emerging evidence that SLC7A11-high tumors may develop a metabolic vulnerability to disulfidptosis (8, 9), which can synergize with immune checkpoint inhibitors to produce enhanced antitumor effects. Collectively, these findings suggest that while SLC7A11 protects against ferroptosis, it simultaneously establishes metabolic dependencies that can be therapeutically exploited to enhance immune recognition. However, the interplay among SLC7A11, ferroptosis/disulfidptosis, and antitumor immunity was not directly tested in this study and warrants functional and *in vivo* validation.

To delineate the upstream regulatory mechanism of SLC7A11 in breast cancer, we verified miR-148b-3p as a novel and potent negative regulator in TNBC cell models (MDA-MB-231 and MDA-MB-468). Accumulating evidence suggests that downregulation of miR-148b-3p suppresses cell proliferation and tumor progression by directly targeting multiple oncogenes, including CCK2R (26) and AMPK (27), across a range of malignancies such as pancreatic and colorectal cancers. Although the molecular mechanisms underlying miR-148b-3p mediated tumorigenesis have been partially characterized, its specific role in breast cancer remains to be elucidated. The results of this study provide the first evidence that miR-148b-3p directly targets the 3’-UTR of SLC7A11. Furthermore, *in vitro* experiments showed that miR-148b-3p inhibition was associated with increased SLC7A11 expression and phenotypes consistent with reduced ferroptosis sensitivity, including increased GSH level and reduced lipid peroxidation. These findings suggest that downregulation of miR-148b-3p promotes resistance to erastin-induced ferroptosis by directly targeting SLC7A11 in TNBC cells, thereby providing a potential mechanistic basis for understanding how ferroptosis sensitivity influences breast cancer cell proliferation and migration. The regulatory landscape of SLC7A11 is complex, involving endoplasmic reticulum stress (30), transcriptional control (31), ubiquitination degradation (31, 32), and as our study reveals, post-transcriptional regulation by miRNAs. These findings position the SLC7A11/miR-148b-3p axis as a candidate regulatory node for ferroptosis modulation in TNBC cell models. Future studies exploring pharmacological targeting of this axis, such as combination strategies with existing ferroptosis inducers, may provide insights into overcoming therapy resistance.

Our study has several limitations. First, the immune-related findings derive from correlative, inference-based metrics, and the immunotherapy-associated analyses rely on heterogeneous public datasets with limited covariate adjustment. Second, mechanistic experiments are conducted in only two TNBC cell models (MDA-MB-231 and MDA-MB-468) and lack *in vivo* validation. Third, no functional immune assays or tumor-immune interaction experiments are performed. The proposed link between ferroptosis and immune susceptibility is not directly tested.

In summary, our integration of immune and ferroptosis analyses and the subsequent validation of miR-148b-3p/SLC7A11 ferroptosis mechanism in TNBC cell models allow us to generate the hypothesis that SLC7A11 may serve as a biomarker of immunotherapy response and a regulator of ferroptosis via miR-148b-3p. Further functional and clinical studies are warranted to confirm the hypothesis across cancer types.

## Data Availability

The raw data supporting the conclusions of this article will be made available by the authors, without undue reservation.
