# Supplementary material for: An integrated analysis of SLC7A11 as a pan-cancer immunotherapeutic biomarker with experimental validation of its regulation by miR-148b-3p in breast cancer
Source: Front Immunol. 2026 May 19;17:1752767. doi: 10.3389/fimmu.2026.1752767 (PMC13226593; doi:10.3389/fimmu.2026.1752767)
Supplement: Supplementary file 1 [file DataSheet1.zip › Supplementary Figures.DOCX]

**Supplementary Figures**

**Figure S1. SLC7A11 expression across TCGA cancer types and subtypes.** The box-and-whisker plot displays the SLC7A11 expression levels (log2 Transcripts Per Million, TPM) specifically within the TCGA dataset. Red boxes represent tumor samples, and blue boxes represent normal or paracancerous tissues. Purple boxes in specific columns (e.g., SKCM) represent metastatic samples. This plot further details expression patterns across molecular subtypes (e.g., BRCA-Basal, BRCA-Her2, BRCA-LumA/B). Statistical significance between groups was evaluated using the Wilcoxon rank-sum test, with adjusted p-values indicated as follows: * *P* < 0.05, ** *P* < 0.01, and *** *P* < 0.001.


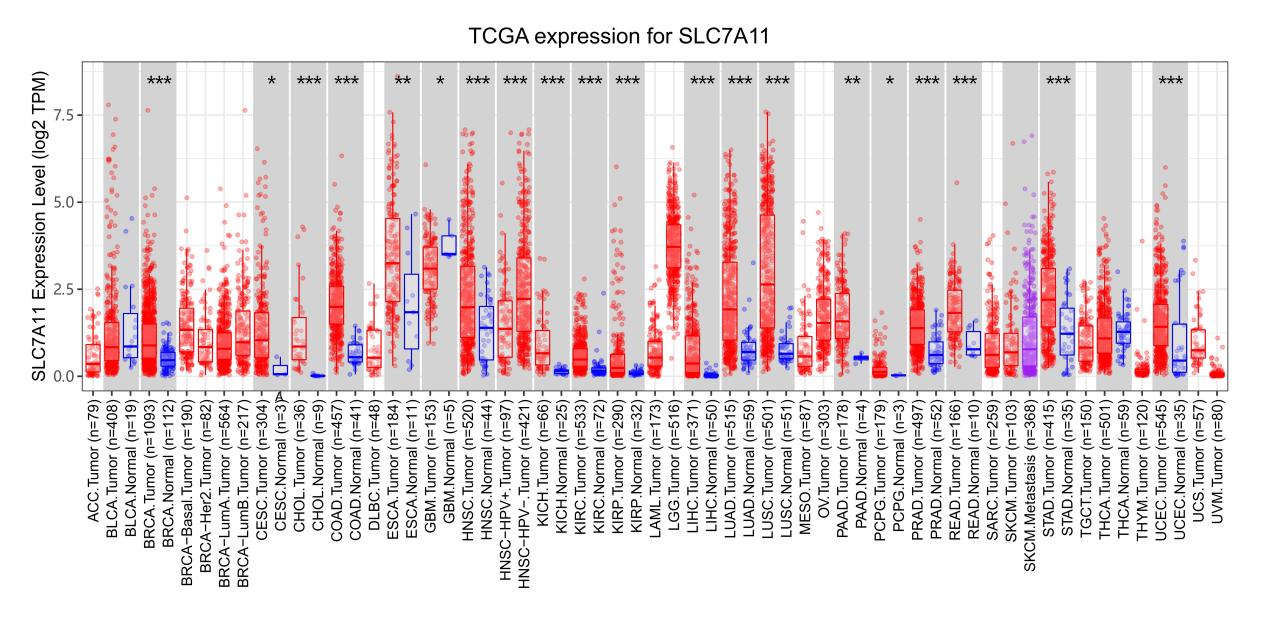


**Figure S2. Differential analysis of SLC7A11 protein levels based on CPTAC data.** Protein expression levels of SLC7A11 in primary tumors and corresponding normal tissues were analyzed using the UALCAN portal, which provides analysis of the Clinical Proteomic Tumor Analysis Consortium (CPTAC) datasets. The y-axis represents the Z-value, which indicates standard deviations from the median across samples for the specific cancer type. The box plots depict the distribution of protein expression: the center line within the box represents the median, while the upper and lower boundaries of the box indicate the 75th and 25th percentiles, respectively. Sample sizes (n) for normal and primary tumor groups are provided below each plot. *** indicates adjusted *P* < 0.001.


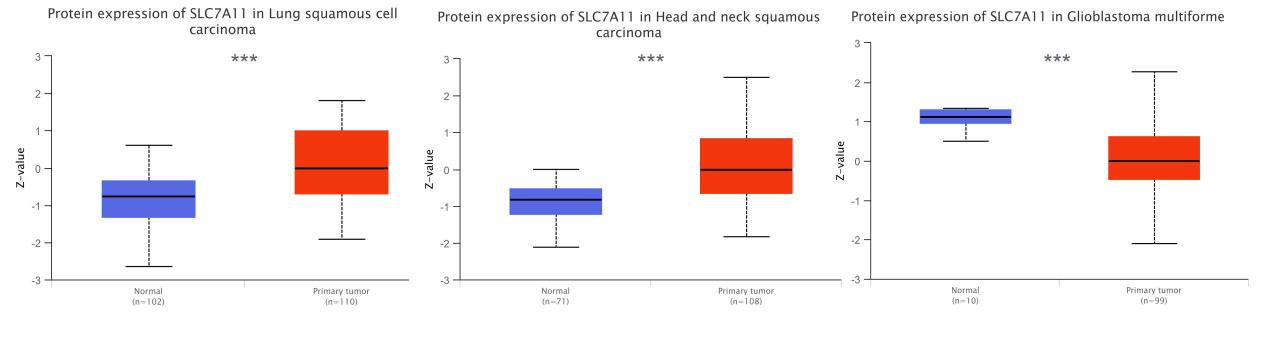


**Figure S3. Correlation of SLC7A11 expression with TMB, MSI, and HRD across pan-cancer.** Lollipop charts illustrate the Pearson correlation between SLC7A11 mRNA expression and three key genomic signatures: (A) Tumor Mutational Burden (TMB), (B) Microsatellite Instability (MSI), and (C) Homologous Recombination Deficiency (HRD) across diverse TCGA cancer types. The horizontal axis represents the Pearson correlation coefficient, where positive values (red lines) indicate a positive correlation and negative values (blue lines) indicate a negative correlation. The size of each dot is proportional to the sample size of the respective cancer cohort. The color gradient represents the statistical significance, ranging from blue to bright red, with redder tones indicating higher significance.


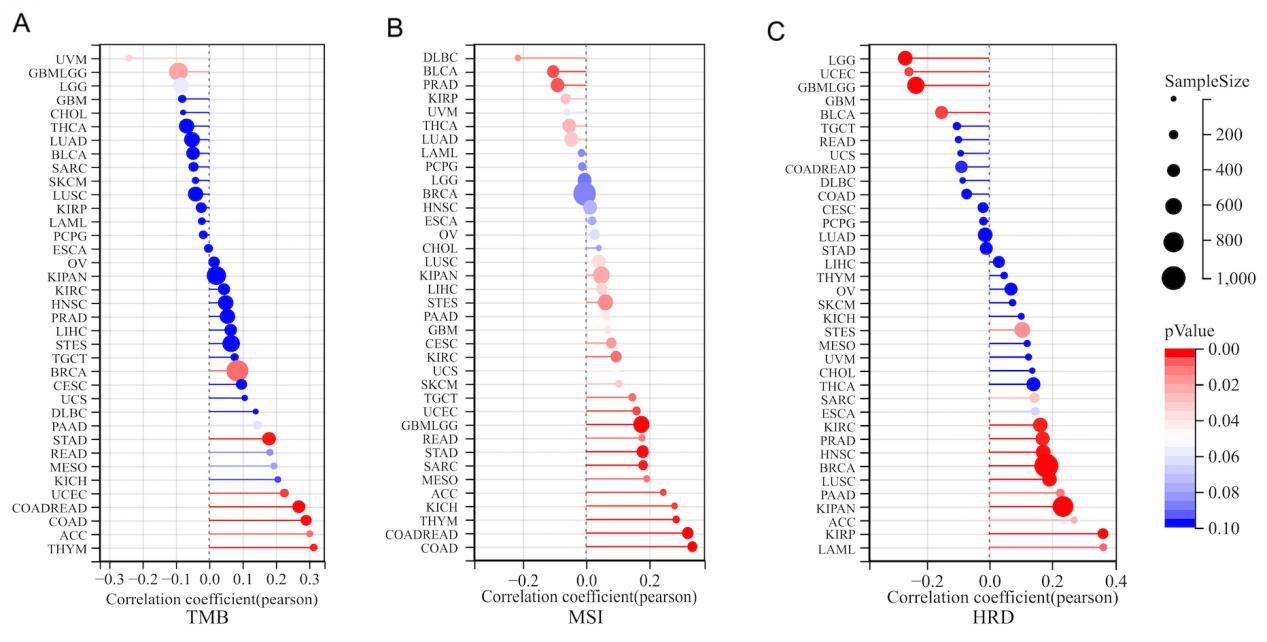


**Figure S4. Association of SLC7A11 expression with DNA repair, stemness, and epigenetic modifications.** (A) Correlation between SLC7A11 and DNA mismatch repair (MMR) genes. The heatmap displays the pan-cancer correlation between SLC7A11 mRNA levels and five core MMR genes. The color of each cell represents the correlation coefficient, while the stars indicate statistical significance. (B) Correlation between SLC7A11 expression and tumor stemness. The lollipop chart illustrates the Pearson correlation between SLC7A11 and the mRNA-based stemness index (mRNAsi). The horizontal axis represents the correlation coefficient, with the size of each dot proportional to the sample size and the color gradient reflecting the p-values. (C) Landscape of SLC7A11 expression and RNA modification regulators. The heatmap summarizes the pan-cancer correlations between SLC7A11 and various RNA modification regulators, including those involved in m1A, m5C, and m6A modifications (categorized as writers, readers, and erasers). The diagonal split in each cell indicates the correlation coefficient (upper left) and the corresponding p-values (lower right). * indicates *P* < 0.05, ** *P* < 0.01, *** *P* < 0.001.


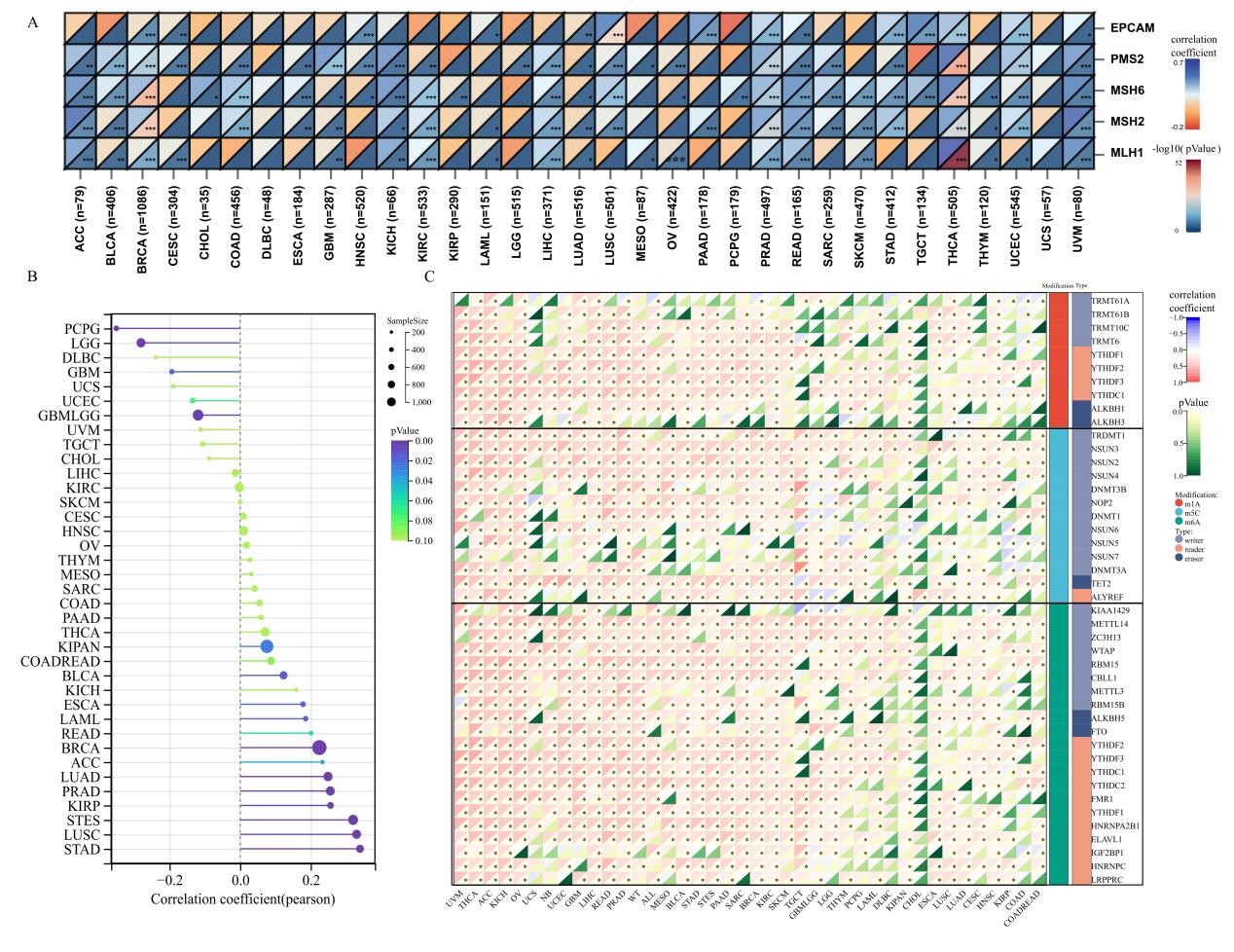


**Figure S5. Association of SLC7A11 expression with immunomodulatory genes and immune infiltration.** (A) Pan-cancer correlation between SLC7A11 and immune cell infiltration. The heatmap illustrates the correlation between SLC7A11 mRNA expression and the infiltration levels of six major immune cell types, including B cells, CD4^+^ T cells, CD8^+^ T cells, neutrophils, macrophages, and dendritic cells (DC) across diverse cancer types. Each cell is diagonally split: the upper-left triangle indicates the Pearson correlation coefficient and the lower-right triangle signifies the statistical significance. (B) Correlation between SLC7A11 and immunomodulators. The heatmap displays the association between SLC7A11 expression and 60 key genes involved in immune checkpoint pathways, including inhibitory (red dots on the right y-axis) and stimulatory (blue dots on the right y-axis) factors. The diagonal split in each cell represents the correlation coefficient (upper left) and the p-values (lower right). Statistical significance is indicated by asterisks: * *P* < 0.05, ** *P* < 0.01, *** *P* < 0.001, **** *P* < 0.0001.


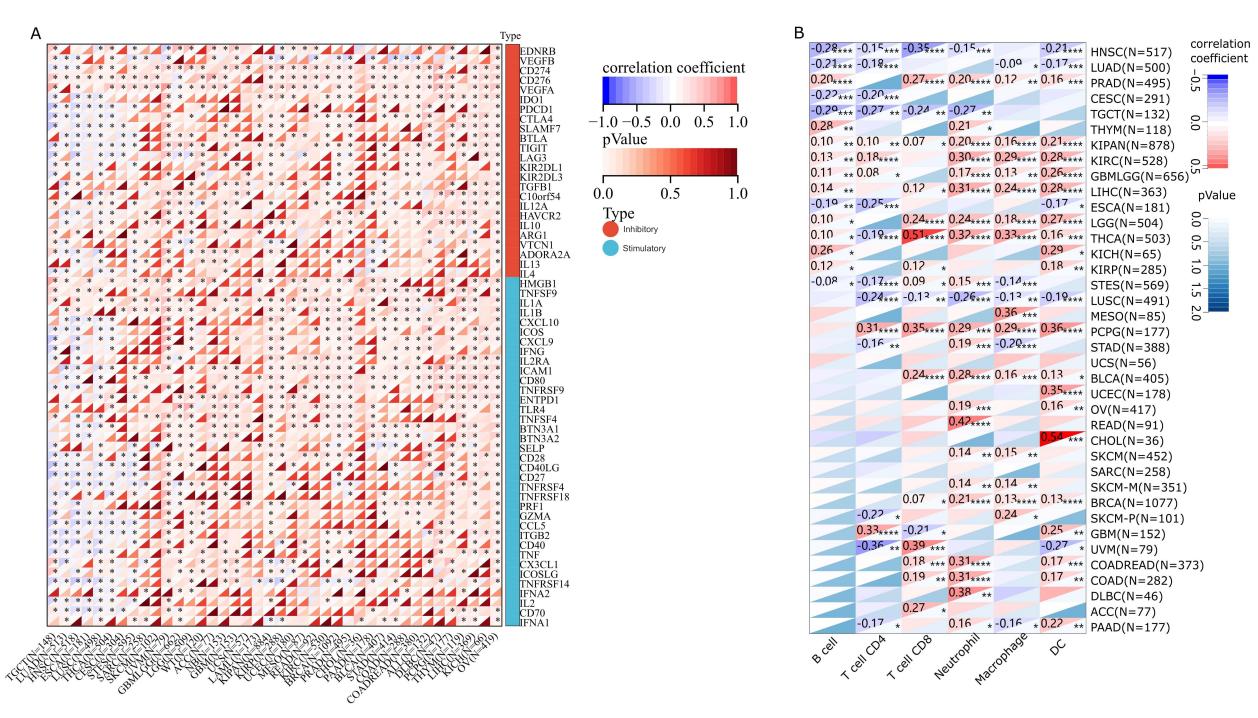


**Figure S6. Correlation between SLC711 expression and immune score in HNSC.** The scatter plot illustrates the association between SLC7A11 mRNA expression and the immune score within the Head and Neck Squamous Cell Carcinoma (HNSC) cohort (N = 517) from the TCGA database. The immune score was calculated using the ESTIMATE algorithm, representing the degree of immune cell infiltration in the tumor microenvironment. Statistical analysis was performed using Pearson correlation, yielding a correlation coefficient of r = -0.35 and a significance level of *P* = 4.7×10^-16^. The regression line (black) and the surrounding 95% confidence interval (gray shaded area) highlight a significant negative correlation. Marginal histograms show the distribution of SLC7A11 expression (top) and ImmuneScore (right) across the patient samples.


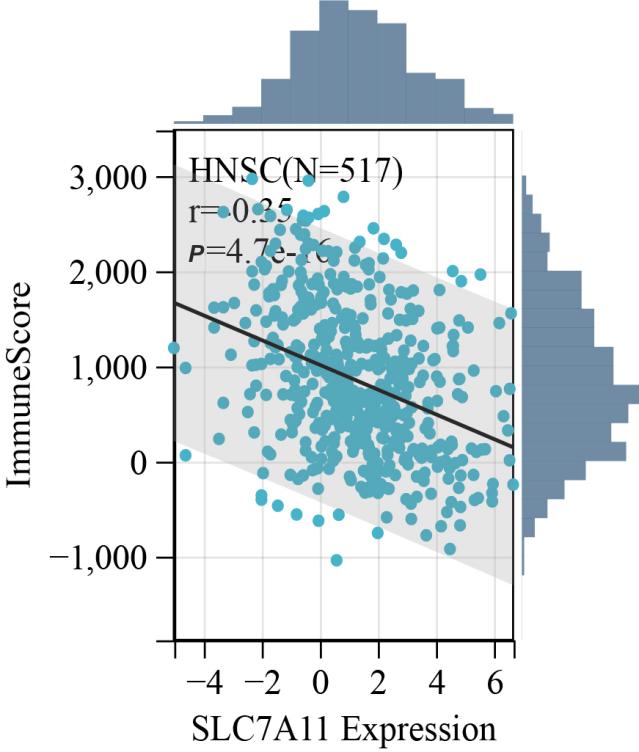


**Figure S7. Prognostic and diagnostic values of SLC7A11 expression across pan-cancer.** (A) Venn diagram illustrating the prognostic significance of SLC7A11. The diagram shows the intersection of cancer types where high SLC7A11 expression is significantly associated with four survival outcomes: Overall Survival (OS), Disease-Specific Survival (DSS), Disease-Free Interval (DFI), and Progression-Free Interval (PFI). (B) Diagnostic potential of SLC7A11 across various tumor types. The circular bar plot displays the Area Under the Curve (AUC) values from Receiver Operating Characteristic (ROC) analyses. The AUC values represent the ability of SLC7A11 expression to distinguish tumor tissues from normal ones. Different colors in the bars represent the gradient of AUC values across the TCGA cohorts.


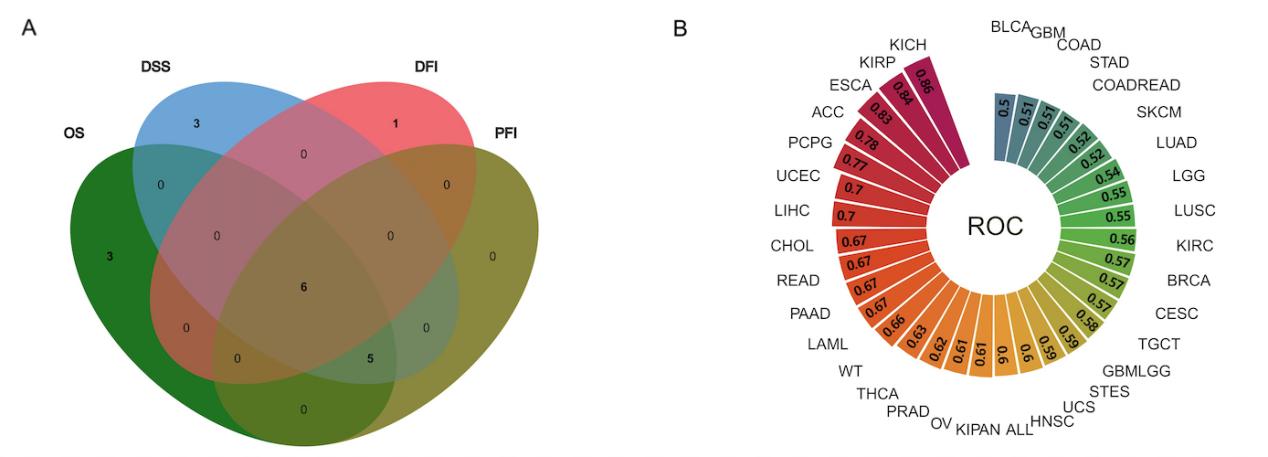


**Figure S8. Uncropped western blot images.** (A) The upper image displays the uncropped western blot for GAPDH, used as the internal loading control. (B) The lower image represents the uncropped western blot for SLC7A11. The protein bands correspond to the samples treated with negative control (NC) inhibitors and miR-148b-3p inhibitors as described in Figure 5C. Molecular weight markers were used to confirm the specific migration of GAPDH (~37 kDa) and SLC7A11 (~55 kDa).

**
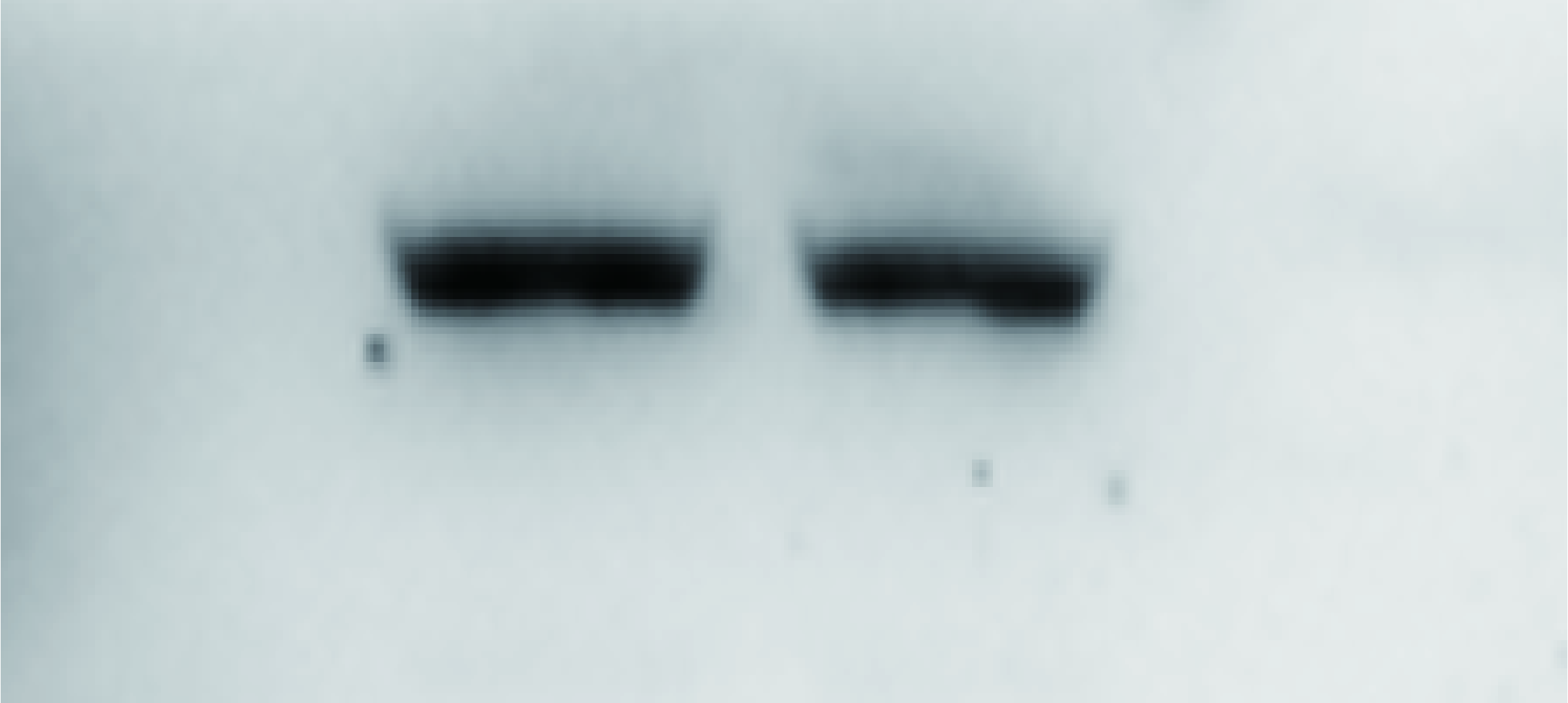
**

**
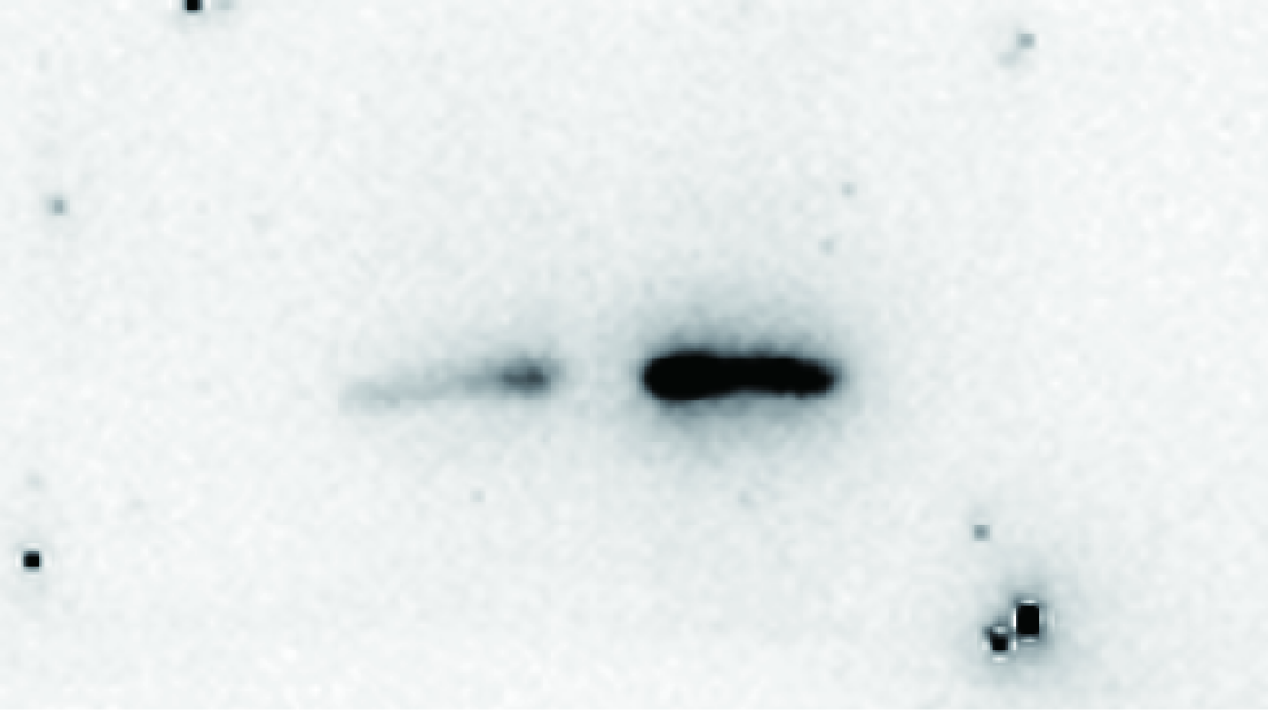
**
